# Induction of the Proinflammatory Chemokine Interleukin-8 Is Regulated by Integrated Stress Response and AP-1 Family Proteins Activated during Coronavirus Infection

**DOI:** 10.3390/ijms22115646

**Published:** 2021-05-26

**Authors:** Qing Chun Zhu, Shumin Li, Li Xia Yuan, Rui Ai Chen, Ding Xiang Liu, To Sing Fung

**Affiliations:** 1 Integrative Microbiology Research Centre, South China Agricultural University, Guangzhou 510642, China; qingchunz2021@163.com (Q.C.Z.); shuminli0312@163.com (S.L.); 13640837315@163.com (L.X.Y.); 2 College of Veterinary Medicine, South China Agricultural University, Guangzhou 510642, China; chensa727@126.com; 3 Zhaoqing Branch, Center of Guangdong Laboratory for Lingnan Modern Agricultural Science and Technology, Zhaoqing 526000, China

**Keywords:** coronavirus, unfolded protein response, integrated stress response, eIF2α, AP-1 family proteins, cJUN and cFOS, proinflammatory cytokine, interleukin-8

## Abstract

Infection induces the production of proinflammatory cytokines and chemokines such as interleukin-8 (IL-8) and IL-6. Although they facilitate local antiviral immunity, their excessive release leads to life-threatening cytokine release syndrome, exemplified by the severe cases of coronavirus disease 2019 (COVID-19) caused by severe acute respiratory syndrome coronavirus 2 (SARS-CoV-2) infection. In this study, we investigated the roles of the integrated stress response (ISR) and activator protein-1 (AP-1) family proteins in regulating coronavirus-induced IL-8 and IL-6 upregulation. The mRNA expression of IL-8 and IL-6 was significantly induced in cells infected with infectious bronchitis virus (IBV), a gammacoronavirus, and porcine epidemic diarrhea virus, an alphacoronavirus. Overexpression of a constitutively active phosphomimetic mutant of eukaryotic translation initiation factor 2α (eIF2α), chemical inhibition of its dephosphorylation, or overexpression of its upstream double-stranded RNA-dependent protein kinase (PKR) significantly enhanced IL-8 mRNA expression in IBV-infected cells. Overexpression of the AP-1 protein cJUN or its upstream kinase also increased the IBV-induced IL-8 mRNA expression, which was synergistically enhanced by overexpression of cFOS. Taken together, this study demonstrated the important regulatory roles of ISR and AP-1 proteins in IL-8 production during coronavirus infection, highlighting the complex interactions between cellular stress pathways and the innate immune response.

## 1. Introduction

Since the start of this century, three animal coronaviruses have crossed the species barrier and caused severe disease in humans. In 2003, the severe acute respiratory syndrome coronavirus (SARS-CoV) that originated in bats caused the SARS outbreak and plunged the world into panic [1,2]. Then the Middle East respiratory syndrome coronavirus (MERS-CoV) emerged in 2012 and caused two regional outbreaks with intermittent sporadic cases [3,4]. The ongoing coronavirus disease 2019 (COVID-19) pandemic is caused by SARS-CoV-2 and has become the most devastating outbreak since the H1N1 influenza in 1918 [5,6]. Therefore, unraveling the mechanisms of coronavirus pathogenesis is a pressing problem with great clinical importance. 

Coronaviruses are a group of enveloped RNA viruses in the family *Coronaviridae* and the order *Nidovirales*. They have single-stranded, non-segmented, positive-sense RNA genomes of 27–32 kilobases [7]. The genome encodes four structural proteins, namely spike (S), small envelope (E), membrane (M), and nucleocapsid (N), as well as 15–16 non-structural proteins (nsps) and some accessory proteins [8]. The avian infectious bronchitis virus (IBV) is a highly contagious gammacoronavirus that causes acute respiratory diseases in chickens, with some nephropathogenic variants also infecting the urinary and reproductive systems. IBV infects both broiler and egg-laying chickens, causing huge economic losses to the global poultry industry [9,10]. The porcine epidemic diarrhea virus (PEDV) is an alphacoronavirus causing highly contagious acute enteritis and fatal watery diarrhea in piglets [11].

The innate immune system is necessary for the initial detection and restriction of viral infection, as well as the subsequent activation of the adaptive immune response. Coronaviruses are recognized by cytosolic and/or endosomal pattern recognition receptors (PRRs), which activate adaptor proteins and downstream pathways. This eventually leads to the activation of critical transcription factors such as nuclear factor kappa light chain enhancer of activated B cells (NF-κB), interferon regulatory factor 3/7 (IRF-3/7), and activator protein 1 (AP-1). These proteins then activate the transcription of type I/III interferons (IFN-I/III) and proinflammatory cytokines/chemokines such as tumor necrosis factor-alpha (TNF-α), interleukin-6 (IL-6), and IL-8. Acting locally, these cytokines/chemokines recruit immune cells and facilitate antiviral responses; but their excessive and uncontrolled release can lead to life-threatening cytokine release syndrome (CRS) that underlies the pathogenesis of severe coronavirus diseases [12,13,14]. Indeed, multiple proinflammatory cytokines have been implicated in the pathogenesis of severe COVID-19 [15]. Among them, high levels of IL-6 and IL-8 were observed in patients with severe or critical COVID-19, correlated with lymphocytopenia that was predictive of disease progression [16]. High levels of IL-6 and IL-8 were also detected in SARS patients [17,18,19] and in some cell lines infected with SARS-CoV [20,21].

The integrated stress response (ISR) is an adaptive pathway activated by eukaryotic cells in response to various stress stimuli. The core event in this pathway is the phosphorylation of eukaryotic translation initiation factor 2α (eIF2α) [22] by one or more of the four known eIF2α kinases: heme regulatory inhibitor (HRI), double-stranded RNA-dependent protein kinase (PKR), general control nonderepressible 2 (GCN2), and PKR-like ER kinase (PERK) [23,24,25,26,27]. The phosphorylated eIF2α suppresses global protein synthesis but several genes become preferentially translated under ISR, including CCAAT-enhancer-binding protein homologous protein (CHOP) and GADD34 (growth arrest and DNA damage-inducible protein 34). CHOP activates apoptosis of the stressed cells, whereas GADD34 is a subunit of protein phosphatase 1 that dephosphorylates eIF2α and reverts the translational block. Significant phosphorylation of PKR and PERK was observed in cells infected with SARS-CoV, MERS-CoV, and IBV [27,28,29]. Previously we have also shown that IBV infection upregulates GADD34 to maintain de novo protein synthesis in the infected cells [30]. Notably, in cells infected with transmissible gastroenteritis virus (TGEV), PERK/eIF2α-mediated translation attenuation reduced the expression of IκBα, thereby activating NF-κB-dependent IFN-I production to suppress TGEV replication [31]. However, it is still uncertain how ISR contributes to the induction of proinflammatory cytokines/chemokines during coronavirus infection.

The AP-1 transcription factors mainly include proteins of the JUN family (cJUN, JUNB, and JUND) and the FOS family (cFOS, FOSB, Fra-1, and Fra-2) [32,33]. AP-1 proteins regulate the transcription of a wide variety of genes involved in numerous cellular functions [34]. Some AP-1 proteins are also phosphorylated and activated by mitogen-activated protein kinases (MAPKs), a group of evolutionarily conserved serine/threonine kinases that include p38, cJUN N-terminal kinase (JNK), and extracellular signal-regulated kinase (ERK) [35]. Significant induction and activation of AP-1 proteins have been observed in coronavirus-infected cells [36,37] and in cells expressing coronavirus structural [38,39] or accessory proteins [40,41,42,43]. Recently we have shown that IBV infection activates both cJUN and cFOS, and their upstream MAPK signaling pathways play vital roles in regulating apoptosis and innate immunity during IBV infection [37,44,45]. However, the functional interactions between individual AP-1 family proteins in the context of coronavirus infection are not fully elucidated.

In this study, we characterized the roles of ISR and AP-1 family proteins in the transcriptional induction of proinflammatory cytokines/chemokines during coronavirus infection. In IBV-infected cells, the induction of IL-8 mRNA was significantly upregulated by the overexpression of eIF2α kinase PKR or a constitutively active mutant of eIF2α, or by the inhibition of eIF2α dephosphorylation. In addition, IBV-induced IL-8 expression was synergistically activated by cJUN and cFOS, with activating signals coming from the upstream MKK7–JNK pathway. Taken together, the ISR and AP-1 family proteins are crucial for the induction of proinflammatory chemokine IL-8 in IBV-infected cells, and may be potential therapeutic targets for immunopathologies associated with coronavirus infections.

## 2. Results

### 2.1. Induction of IL-8 and IL-6 Was Detected in Cells Infected with Gammacoronavirus IBV and Alphacoronavirus PEDV

**Figure 1:**
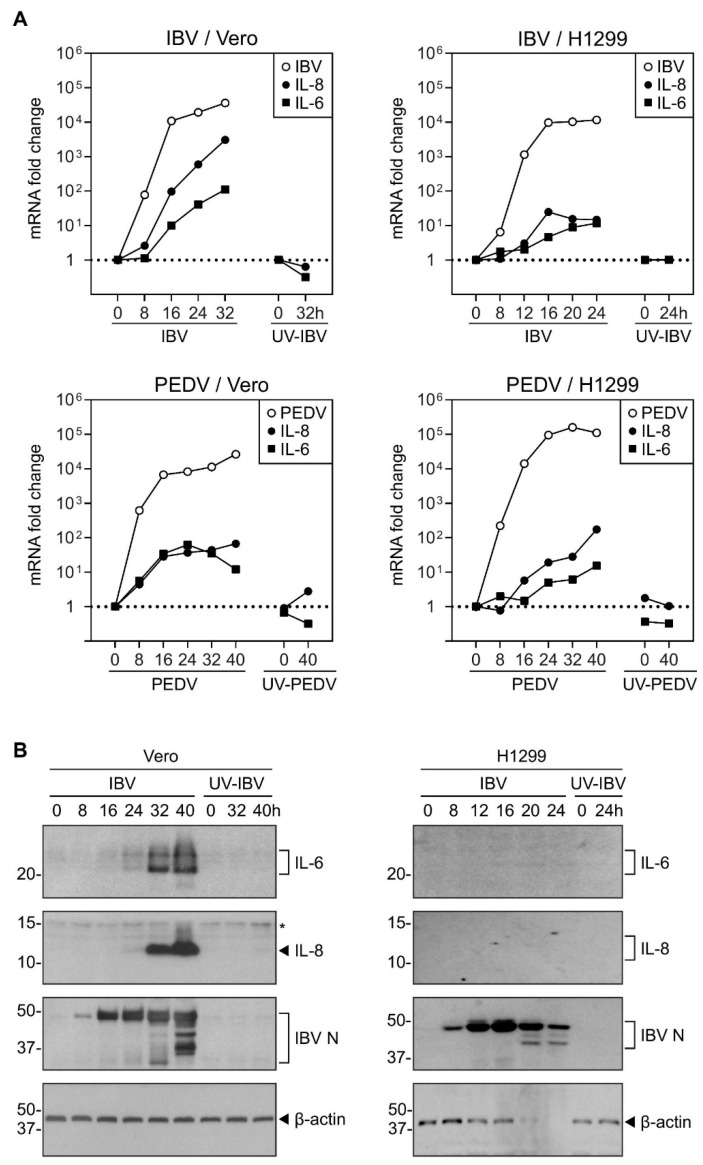
Upregulation of IL-8 mRNAs and proteins during IBV and PEDV infection. (**A**) H1299 and Vero cells were infected with IBV and PEDV at MOI~2 or mock-treated with UV-inactivated viruses. Cell were harvested at the indicated time points and total RNA samples were extracted for RT-qPCR. The levels of IBV genomic RNA (IBV) and PEDV genomic RNA (PEDV), and the mRNA levels of IL-8 and IL-6 were determined by the ΔΔCt method using the GAPDH mRNA of the virus-infected 0 hpi sample for normalization. The experiment was repeated three times with similar results, and the result of one representative experiment is shown. (**B**) Vero and H1299 cells were infected with IBV at MOI~2 or mock-treated with UV-inactivated IBV. Cell lysates were harvested at the indicated time points and subjected to Western blot analysis using the indicated antibodies. Beta-actin was included as the loading control. Sizes of protein ladders, in kDa, are indicated on the left. The experiment was repeated three times with similar results, and the result of one representative experiment is shown. Asterisk (*) indicates the nonspecific band detected by the IL-8 antibody.

To determine the induction of IL-8 during coronavirus infection, Vero and H1299 cells were infected with IBV and PEDV at MOI~2, respectively. Cells incubated with the same amounts of UV-inactivated viruses, UV-IBV and UV-PEDV, were also included as mock-treated controls. In IBV-infected Vero cells, the IL-8 mRNA level was significantly elevated starting from 16 hpi, and was induced by over 3-log at 32 hpi (Figure 1A). Due to the extensive RNA degradation, the RNA sample at 40 hpi was not analyzed. Although to a lesser extent, similar induction of IL-8 mRNA was also observed in IBV-infected H1299 cells as well as in PEDV-infected Vero and H1299 cells (Figure 1A). No induction of IL-8 was observed in cells incubated with UV-IBV and UV-PEDV, respectively (Figure 1A). 

The expression of IL-8 protein was also determined. In IBV-infected Vero cells, IL-8 protein was not detected at 0, 8, or 16 hpi, but accumulated to high levels from 32 to 40 hpi, whereas no IL-8 protein was detected in cells incubated with UV-IBV (Figure 1B). However, the IL-8 protein was not detected in IBV-infected H1299 cells (Figure 1B), probably due to the lower IL-8 induction at the mRNA levels in these cells. 

The same infected cells were also used to detect the expression of IL-6. In IBV-infected Vero cells, IL-6 mRNA levels increased significantly from 16 hpi, and were induced by close to 2-log at 32 hpi (Figure 1A). Similar IL-6 mRNA induction was also observed in IBV-infected H1299 cells and PEDV-infected Vero and H1299 cells (Figure 1A). IL-6 induction was not observed in cells incubated with UV-IBV and UV-PEDV, respectively (Figure 1A).

In IBV-infected Vero cells, IL-6 protein was not detected at 0, 8, or 16 hpi, but accumulated to high levels at 32 to 40 hpi, while IL-6 protein was not detected in cells incubated with UV-IBV (Figure 1B). Again, IL-6 protein was not detected in IBV-infected H1299 cells (Figure 1B). 

In summary, these data indicate that IL-6 and IL-8 are significantly induced late in the coronavirus infection cycle.

### 2.2. Overexpression of PKR UpRegulated the IBV-Induced IL-8 Expression

**Figure 2:**
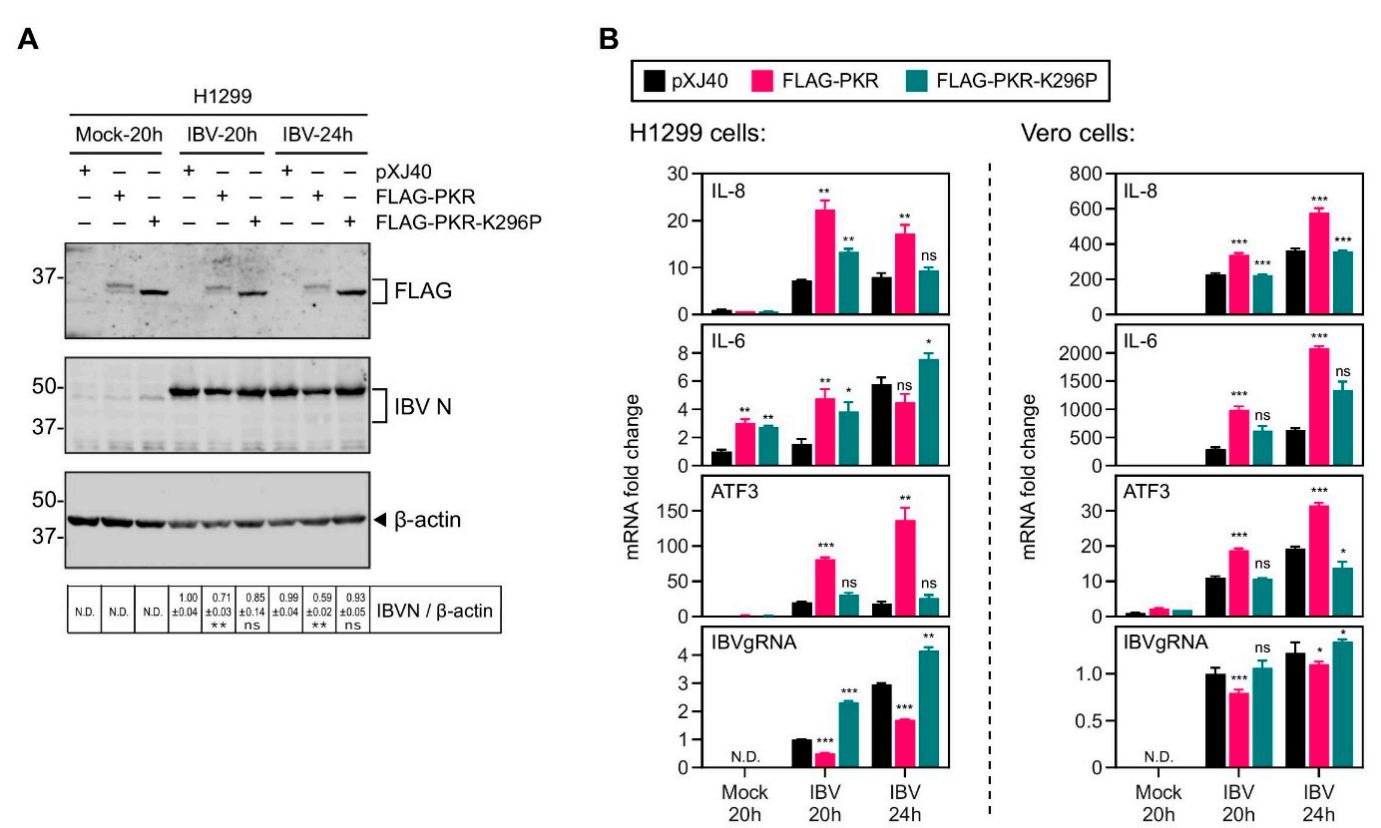
Overexpression of PKR increased the induction of IL-8 mRNA in cells infected with IBV. (**A**) H1299 cells were transfected with *pXJ40*, *pXJ40-FLAG-PKR*, or *pXJ40-FLAG-PKR-K296P*, before being infected with IBV at MOI~2 or mock infected (M). Cell were harvested at the indicated time points and subjected to Western blot analysis using the indicated antibodies. Beta-actin was included as the loading control. Sizes of protein ladders in kDa are indicated on the left. Significance levels are presented by the *p* value (**, *p* < 0.01; ns, non-significant). (**B**) Total RNA samples were extracted from cells in (**A**) and subjected to RT-qPCR. The levels of IL-8, IL-6, ATF3, and IBV genomic RNA were determined by the ΔΔCt method using the GAPDH mRNA of the pXJ40-transfected, 20 h post mock-infected sample for normalization. Vero cells were transfected, infected, and analyzed similarly. The experiment was repeated three times with similar results, and the result of one representative experiment is shown. Significance levels were presented by the *p*-value (ns, non-significant; *, *p* < 0.05; **, *p* < 0.01; ***, *p* < 0.001; N.D., non-determined).

We have previously demonstrated that the PERK/PKR–eIF2α–ATF3 pathway is activated by IBV infection [27]. To examine if this pathway is involved in IBV-induced IL-8 and IL-6 expression, H1299 cells were transfected with FLAG-tagged wild type PKR and its catalytically inactive mutant K296P, respectively, before being infected with IBV. The overexpressed wild type PKR was detected as a doublet in both IBV-infected and mock-treated cells, representing the autophosphorylated (upper band) and non-phosphorylated (lower band) forms of the protein (Figure 2A). Only a single band comigrating with the non-phosphorylated form of PKR was detected in cells transfected with K296P (Figure 2A), indicating that the overexpressed wild type PKR may be functionally active, while the K296P mutation indeed blocked the autophosphorylation of the protein. It was also noted that the level of overexpressed PKR protein was markedly lower than K296 regardless of IBV infection, suggesting a more rapid turnover rate of the activated PKR as well as some negative feedback regulations. Interestingly, a moderately lower level of IBV N protein was detected in PKR-overexpressing cells, compared with the vector control or K296-overexpressing cells (Figure 2A).

Compared with the vector control, overexpression of PKR but not K296P resulted in a significant increase of IL-8 and IL-6 mRNA induction in IBV-infected H1299 cells (Figure 2B). As a downstream protein induced in the PKR–eIF2α pathway, ATF3 mRNA levels were also markedly enhanced in PKR-overexpressing H1299 cells (Figure 2B), further supporting that the ectopic PKR protein is functionally active. In contrast, the levels of IBV genomic RNA were reduced by half in PKR-overexpressing H1299 cells, but only moderately increased in K296P-overexpressing H1299 cells (Figure 2B). The observed inhibitory effect of PKR on IBV replication was presumably due to its activation of eIF2α and the resultant translation attenuation. The same experiment was performed in Vero cells, and the mRNA levels of IL-8, IL-6, and ATF3 induced by IBV infection were further increased in PKR-overexpressing cells, whereas the levels of IBV genomic RNA were marginally reduced (Figure 2B). Taken together, these data suggest that the kinase activity of PKR contributed to IL-6 and IL-8 induction during IBV infection. 

### 2.3. Overexpression of the Constitutively Active eIF2α Increased the IL-8 Induction by IBV Infection

**Figure 3:**
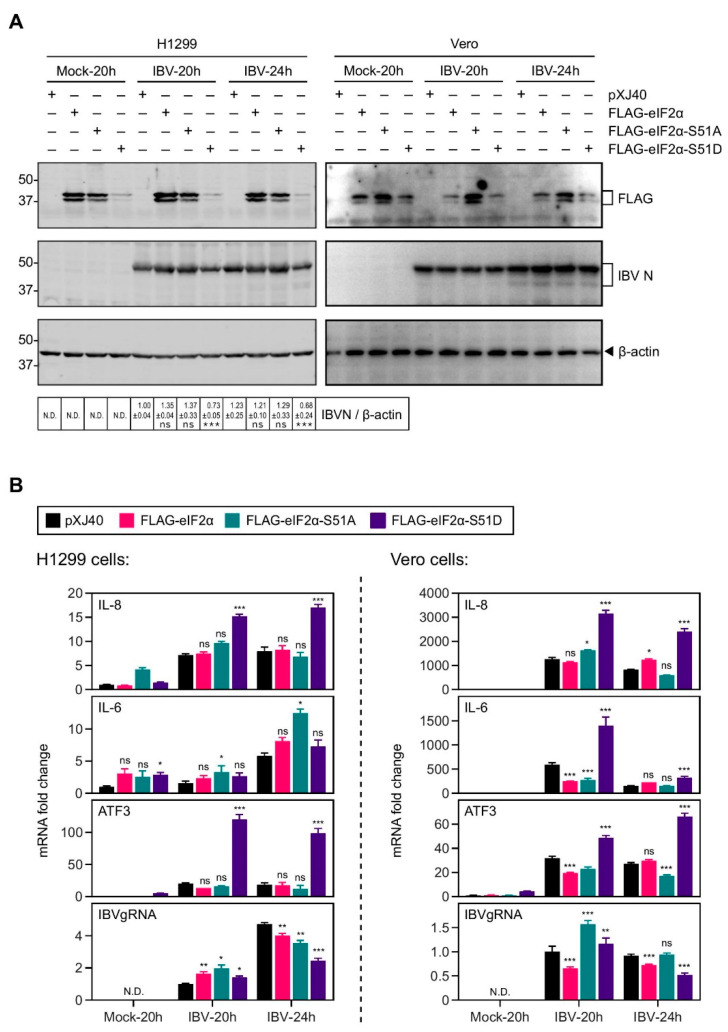
Overexpression of constitutively active eIF2α increased the induction of IL-8 mRNA in cells infected with IBV. (**A**) H1299 and Vero cells were transfected with *pXJ40, pXJ40-FLAG-eIF2α, pXJ40-FLAG-eIF2α-S51A*, or *pXJ40-FLAG-eIF2α-S51D*, before being infected with IBV at MOI~2 or mock infected (M). Cell were harvested at the indicated time points and subjected to Western blot analysis using the indicated antibodies. Beta-actin was included as the loading control. Sizes of protein ladders in kDa are indicated on the left. Significance levels are presented by the *p* value (***, *p* < 0.001; ns, non-significant). (**B**) Total RNA samples were extracted from cells in (**A**) and subjected to RT-qPCR. The levels of IL-8, IL-6, ATF3, and IBV genomic RNA were determined by the ΔΔCt method using the GAPDH mRNA of the pXJ40-transfected, 20 h post mock-infected sample for normalization. The experiment was repeated three times with similar results, and the result of one representative experiment is shown. Significance levels were presented by the *p*-value (ns, non-significant; *, *p* < 0.05; **, *p* < 0.01; ***, *p* < 0.001; N.D., non-determined).

We next analyzed the involvement of eIF2α in IBV-induced IL-8 and IL-6 expression. H1299 cells were transfected with the FLAG-tagged wild type eIF2α, a phosphorylation site mutant S51A, and a constitutively active phosphomimetic mutant S51D, respectively. Similar to PKR, the level of overexpressed S51D protein was much lower than eIF2α or S51A, suggesting potential negative feedback regulations (Figure 3A). In addition, overexpression of S51D resulted in a moderate reduction of IBV genomic RNA replication and N protein translation, presumably due to the translation attenuation (Figure 3A,B).

As shown in Figure 3B, overexpression of the phosphomimetic mutant S51D resulted in a ~2-fold increase in the IBV-induced IL-8 mRNA expression in both H1299 and Vero cells (Figure 3B). Transcription of ATF3 was also significantly increased in the S51D-transfected cells, suggesting that overexpression of S51D effectively activated downstream pathways. However, this was not the case for IL-6 mRNA expression, which was not consistently regulated by S51D overexpression. Taken together, these data suggest that phosphorylation of eIF2α was actively involved in the upregulation of IL-8 during IBV infection.

### 2.4. Inhibition of eIF2α Dephosphorylation Increased the Induction of IL-8 mRNA by IBV Infection

**Figure 4:**
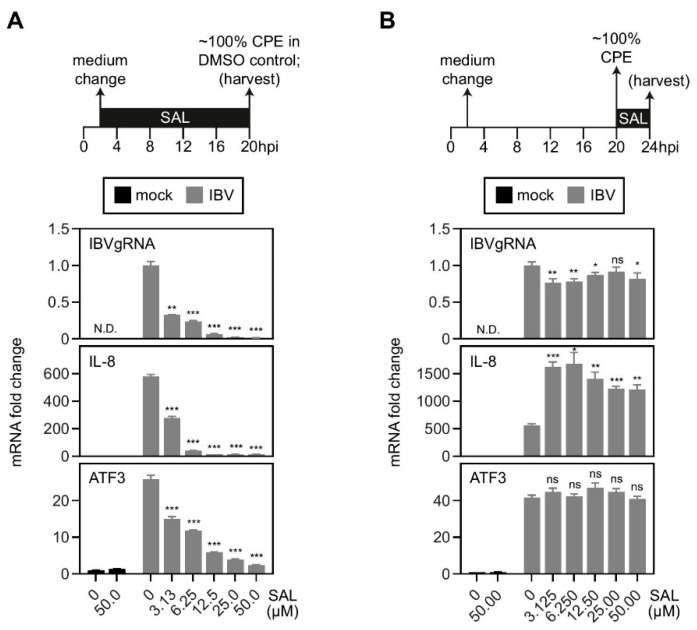
Treatment of eIF2α phosphatase inhibitor, salubrinal, increased the induction of IL-8 mRNA in cells infected with IBV. (**A**) H1299 cells were infected with IBV at MOI~2 or mock infected. Two hours after adsorption, culture medium was changed and cells were treated with salubrinal at the indicated concentrations or the same volume of DMSO. Cells were harvested at 20 hpi and total RNA samples were extracted for RT-qPCR. The levels of IL-8, ATF3, and IBV genomic RNA were determined by the ΔΔCt method using the GAPDH mRNA of the DMSO-treated and mock-infected sample for normalization. The experiment was repeated three times with similar results, and the result of one representative experiment is shown. Significance levels were presented by the *p*-value (**, *p* < 0.01; ***, *p* < 0.001; N.D., non-determined). (**B**) H1299 cells were infected with IBV at MOI~2 or mock infected. Culture medium was changed two hours after adsorption. When 100% CPE was observed in the infected cells at 20 hpi, cells were treated with salubrinal at the indicated concentrations or the same volume of DMSO for 4 h. Cells were harvested and RT-qPCR was performed as in (**A**). The experiment was repeated three times with similar results, and the result of one representative experiment is shown. Significance levels were presented by the *p*-value (ns, non-significant; *, *p* < 0.05; **, *p* < 0.01; ***, *p* < 0.001; N.D., non-determined).

To further validate the role of eIF2α phosphorylation, we used salubrinal (SAL), a chemical known to inhibit PP1, thereby blocking the dephosphorylation of eIF2α. At first, cells were treated with SAL from 2 hpi to 20 hpi. As shown in Figure 4A, IBV genomic RNA replication was significantly suppressed in SAL-treated cells in a dosage-dependent manner, suggesting a potent antiviral activity of SAL against IBV infection. As a consequence of the reduced IBV replication, the mRNA levels of IL-8 and ATF3 were also reduced with increasing SAL concentrations (Figure 4A).

To minimize the inhibitory effect of SAL on IBV replication, in the second set of experiments, cells were treated with SAL from 20 hpi (~100% CPE) to 24 hpi. No significant dosage-dependent inhibition on the levels of IBV genomic RNA replication was observed, presumably because IBV genome replication had plateaued before 20 hpi (Figure 4B). Notably, IBV-induced IL-8 mRNA expression was significantly higher in SAL-treated cells compared with the DMSO-treated control, with a ~3-fold increase even in cells treated with 3.125 µM SAL. In sharp contrast, SAL treatment did not affect ATF3 mRNA levels, suggesting that treatment of cells with SAL for 4 h was not sufficient to activate the ATF3 transcription. It also suggests that the phospho-eIF2α-mediated IL-8 upregulation was not dependent on ATF3.

### 2.5. cJUN and cFOS Play a Synergistic Role in the IL-8 Induction during IBV Infection

**Figure 5:**
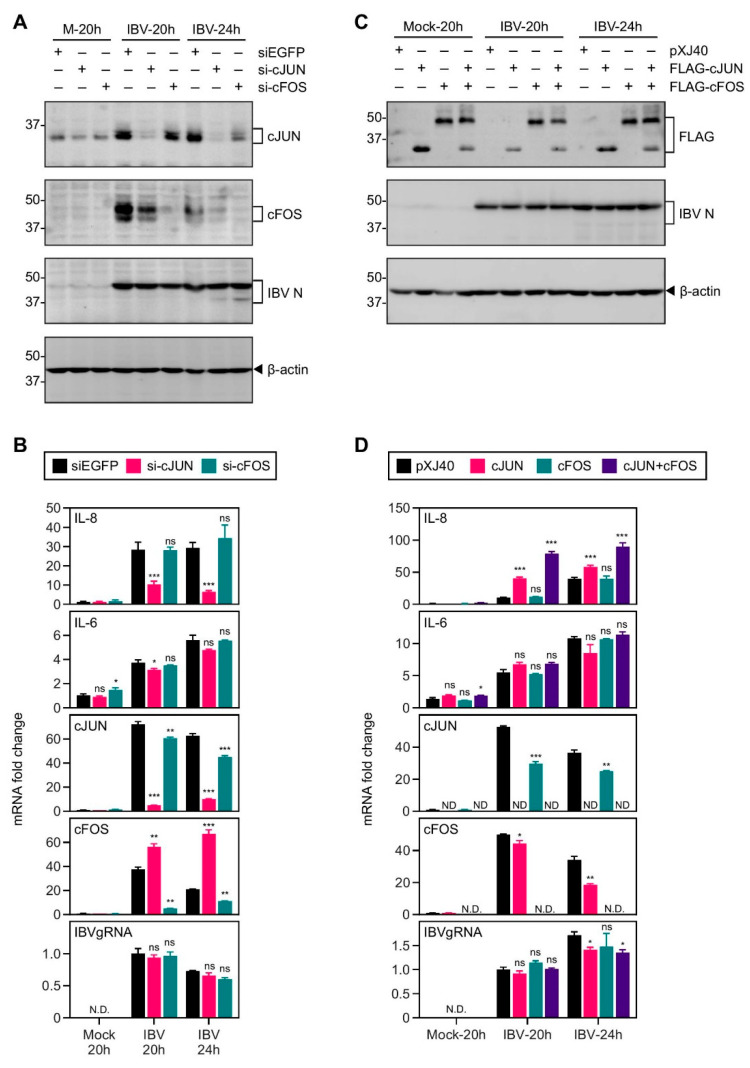
cJUN and cFOS synergistically induced IL-8 mRNA expression in cells infected with IBV. (**A**) H1299 cells were transfected with siEGFP, si-cJUN, or si-cFOS, before being infected with IBV at MOI~2 or mock infected (M). Cell were harvested at the indicated time points and subjected to Western blot analysis using the indicated antibodies. Beta-actin was included as the loading control. Sizes of protein ladders in kDa are indicated on the left. (**B**) Total RNA samples were extracted from cells in (A) and subjected to RT-qPCR. The levels of IL-8, IL-6,cJUN, cFOS, and IBV genomic RNA were determined by the ΔΔCt method using the GAPDH mRNA of the siEGFP-transfected, 20 h post mock-infected sample for normalization. The experiment was repeated three times with similar results, and the result of one representative experiment is shown. Significance levels were presented by the *p*-value (ns, non-significant; *, *p* < 0.05; **, *p* < 0.01; ***, *p* < 0.001; N.D., non-determined). (**C**) H1299 cells were transfected with *pXJ40, pXJ40-FLAG-cJUN, pXJ40-FLAG-cFOS*, or an equal molar mixture of *pXJ40-FLAG-cJUN* and *pXJ40-FLAG-cFOS*, before being infected with IBV at MOI~2 or mock infected (M). Cells were harvested and Western blot analysis using the indicated antibodies. Beta-actin was included as the loading control. Sizes of protein ladders in kDa are indicated on the left. (**D**) Cell lysates in (C) were subjected to RT-qPCR as in (B). The levels of IL-8, IL-6, cJUN, cFOS, and IBV genomic RNA were determined by the ΔΔCt method using the GAPDH mRNA of the pXJ40-transfected, 20 h post mock-infected sample for normalization. The experiment was repeated three times with similar results, and the result of one representative experiment is shown. Significance levels were presented by the *p*-value (ns, non-significant; *, *p* < 0.05; **, *p* < 0.01; ***, *p* < 0.001; N.D., non-determined).

We have previously demonstrated that activation of the MKK7–JNK–cJUN pathway regulates the induction of apoptosis in IBV-infected cells [37]. IBV infection was also shown to upregulate cFOS, which plays a role in the regulation of IBV-induced apoptosis and cytokine induction [37]. To further determine the involvement of cJUN and its homolog cFOS in IBV-induced IL-8 and IL-6 expression, H1299 cells were transfected with siRNA targeting EGFP (negative control), cJUN, and cFOS, before being infected with IBV. As shown in Figure 5A,B, IBV infection led to a significant induction of cJUN and cFOS at both mRNA and protein levels, which was largely diminished in cells transfected with the respective siRNA. Knockdown of cJUN or cFOS did not significantly affect IBV replication, as determined by the similar levels of IBV N protein and IBV genomic RNA, compared with the siEGFP control. Interestingly, IBV-induced IL-8 mRNA expression was markedly reduced in the cJUN-knockdown cells, but remained comparable to the siEGFP control in the cFOS-knockdown cells (Figure 5B). IBV-induced IL-6 mRNA expression was not significantly affected by the silencing of cJUN or cFOS. These data suggests that cJUN, but not cFOS, was essential for the induction of IL-8 mRNA in IBV-infected cells.

To complement the knockdown experiment, H1299 cells were transfected with the FLAG-tagged cJUN or cFOS, or co-transfected with both. As shown in Figure 5C,D, overexpression of cJUN, cFOS, and cJUN/cFOS, respectively, did not significantly affect the IBV genome replication or the synthesis of IBV N protein. Consistent with the knockdown data, overexpression of cJUN, but not cFOS, significantly increased IBV-induced IL-8 mRNA levels, but had no effect on IL-6 mRNA expression, compared with the vector control (Figure 5D). Notably, compared with that in cells transfected with cJUN only, co-transfection of cJUN and cFOS further increased the IBV-induced IL-8 mRNA expression (Figure 5D). Taken together, these data suggest that cJUN directly promoted IL-8 induction in IBV-infected cells, while cFOS served a supportive role presumably by synergistically enhancing the transactivational activity of cJUN.

### 2.6. Activation of the MKK7–JNK–cJUN Pathway Promoted the IBV-Induced IL-8 mRNA Expression

**Figure 6:**
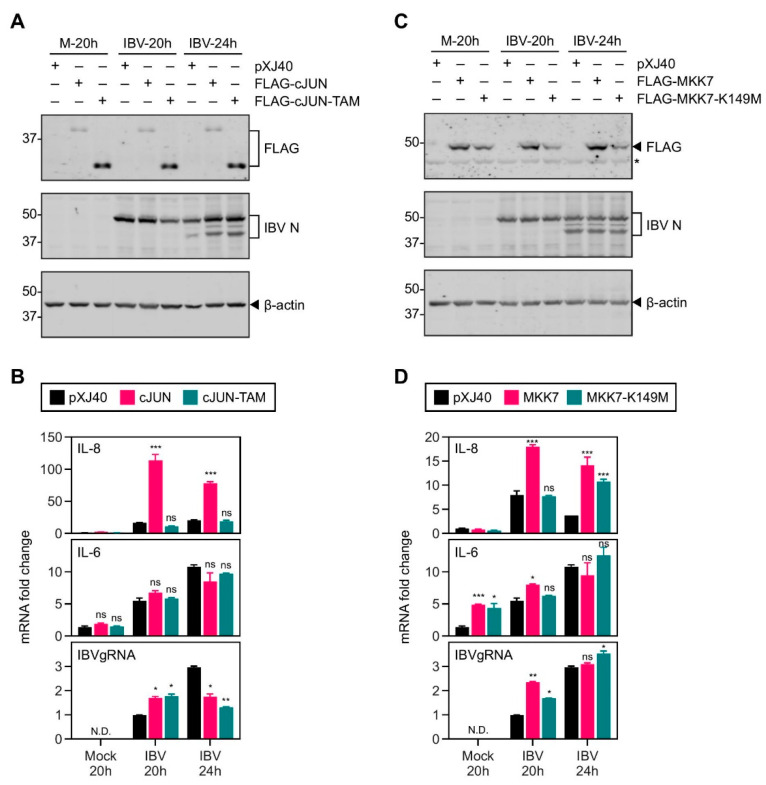
Activation of the MKK7-JNK-cJUN pathway promoted the induction of IL-8 mRNA in cells infected with IBV. (**A**) H1299 cells were transfected with *pXJ40*, *pXJ40-FLAG-cJUN*, or *pXJ40-FLAG-cJUN-TAM*, before being infected with IBV at MOI~2 or mock infected (M). Cell were harvested at the indicated time points and subjected to Western blot analysis using the indicated antibodies. Beta-actin was included as the loading control. Sizes of protein ladders in kDa are indicated on the left. (**B**) Total RNA samples were extracted from cells in (**A**) and subjected to RT-qPCR. The mRNA level of IL-8 and IL-6 was determined by the ΔΔCt method using the GAPDH mRNA of the pXJ40-transfected, 20 h post mock-infected sample for normalization. The experiment was repeated three times with similar results, and the result of one representative experiment is shown. Significance levels were presented by the p-value (ns, non-significant; *, *p* < 0.05; **, *p* < 0.01; ***, *p* < 0.001; N.D., non-determined). (**C**) H1299 cells were transfected with *pXJ40*, *pXJ40-FLAG-MKK7*, or *pXJ40-FLAG-MKK7-K149M*, before being infected with IBV at MOI~2 or mock-infected (M). Cells were harvested and Western blot analysis was performed as in (**A**). (**D**) Cell lysates in (**C**) were subjected to RT-qPCR as in (**B**). The experiment was repeated three times with similar results, and the result of one representative experiment is shown. Significance levels were presented by the *p*-value (ns, non-significant; *, *p* < 0.05; **, *p* < 0.01; ***, *p* < 0.001; N.D., non-determined).

To further validate the functional involvement of cJUN in IBV-induced IL-8 expression, H1299 cells were transfected with the FLAG-tagged cJUN and its transactivation mutant (TAM), respectively. The molecular weight of cJUN is 35.7 kDa. In the transactivation mutant(TAM), amino acids 3–122 spanning the transactivation domain were deleted, resulting in a truncated protein with a size of 22.6 kDa. This was revealed by the smaller protein band of cJUN-TAM in the Western blot. Compared with the vector control, overexpression of either cJUN or TAM did not markedly affect IBV replication, as indicated by the detection of comparable levels of IBV N protein (Figure 6A). Overexpression of cJUN, but not TAM, significantly enhanced the IBV-induced IL-8 (but not IL-6) mRNA expression, supporting the essential function of the transactivational activity of cJUN in the induction of IL-8 during IBV infection (Figure 6B).

As overexpression of MKK7, but not its ATP-binding defective mutant K149M, was shown to increase the JNK and cJUN phosphorylation induced by IBV infection [37], the involvement of this kinase in cJUN-mediated IL-8 induction during IBV infection was then investigated. H1299 cells were transfected with the FLAG-tagged MKK7 and K149M mutant, respectively. The replication of IBV was not significantly affected by the overexpression of either MKK7 or K149M, but the induction of IL-8 (but not IL-6) mRNA was further increased in cells overexpressing MKK7 (Figure 6C,D). These data indicate that the activation of the MKK7–JNK–cJUN pathway contributes to the induction of IL-8 mRNA during IBV infection.

## 3. Discussions

**Figure 7:**
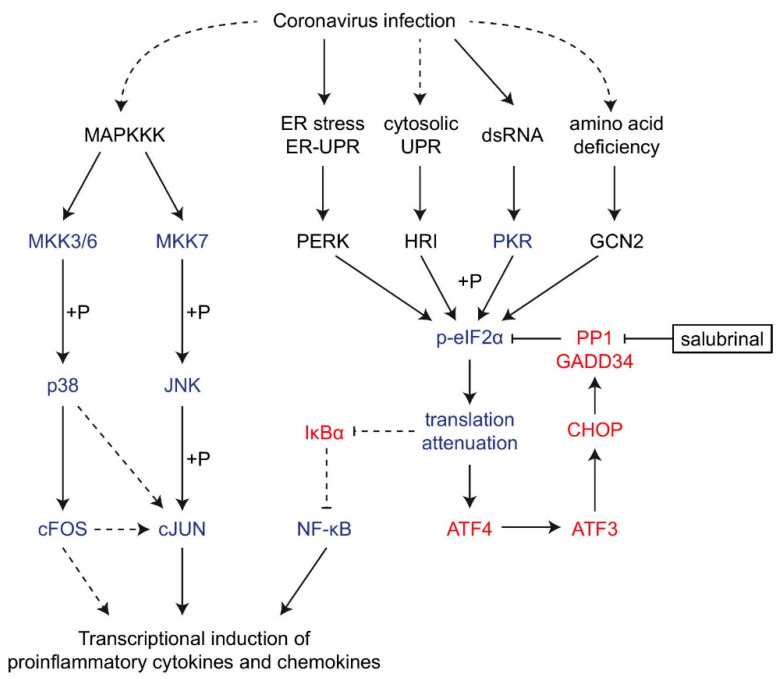
Diagram illustrating the current working model. The working model showing the induction of proinflammatory cytokines and chemokines by integrated stress response and AP-1 family proteins during coronavirus infection. Pointed and blunt arrows denote activation and suppression, respectively. Dotted lines denote processes that are not fully characterized. “+P” denotes phosphorylation.

The innate immune system is the host’s first line of defense against pathogens, but the excessive production of proinflammatory cytokines and chemokines is considered to be the main mediator in the pathogenesis of coronaviruses [6]. In this study, we have shown that the ISR and the AP-1 proteins contribute significantly to the transcriptional induction of IL-8 mRNA during IBV infection (Figure 7). These findings expanded our understanding of the complex mechanisms regulating the innate immune response during coronavirus infection, and provided new insights into the immunopathologies associated with severe coronavirus diseases.

Previous studies have suggested the involvement of cellular stress response in regulating coronavirus replication and the production of interferons and cytokines during infection. For example, the accessory protein 4a of MERS-CoV sequesters dsRNA and suppresses PKR-dependent antiviral stress responses [29], whereas the 4b protein has phosphodiesterase activity that antagonizes the host oligoadenylate synthetase (OAS)-RNase L antiviral pathway [46]. As a result, MERS-CoV lacking the genes for accessory proteins 4a and 4b (MERS-CoV-Δp4) replicates less efficiently than MERS-CoV in cell culture [47]. Notably, deletion of 4a results in increased interferon lambda (IFNL1) expression, but it does not result in robust activation of PKR or the OAS-RNase L pathway [48], suggesting that other proteins encoded by MERS-CoV also contribute to the suppression of innate immune response. 

In terms of proinflammatory cytokines, MHV infection and overexpression of MHV spike protein activated the ER stress response and upregulated the production of IL-8 [49]. IL-8 was also induced in cells infected with a baculovirus displaying SARS-CoV spike protein, and mutation analysis of the IL-8 promoter demonstrated that the AP-1 binding site was crucial to SARS-CoV spike-induced IL-8 production [50]. Previously, we have also shown that IBV infection activates the ER stress, unfolded protein response [27,51], as well as the p38, ERK, and JNK MAPK signaling pathways [37,44]. Among them, the p38 pathway contributed significantly to IBV-induced IL-6 and IL-8 transcription [44]. In this study, The induction of IL-6 and IL-8 is higher in IBV-infected Vero cells than in IBV-infected H1299 cells, which may be due to the following two factors. First, the IBV-p65 strain used in this study was obtained by passaging the IBV Beaudette strain 65 times in the interferon-deficient Vero cells. The replication of IBV-p65 is slightly more efficient in Vero cells than in H1299 cells, which was revealed by the slightly higher levels of IBV genomic RNA and IBV N protein, particularly in the late stages of infection. Second, previous studies have established a strong association between p53 expression and IL-8 mRNA expression in non-small-cell lung carcinoma [52]. Mechanistically, p53 may directly bind to the promoter sequences of IL-6 and IL-8 [53], or it may upregulate Toll-like receptors (TLRs) and enhance TLR-dependent production of pro-inflammatory cytokines [54]. As H1299 is a p53-deficient cell line, the induction of IL-6 and IL-8 after IBV infection may be markedly compromised. In fact, differential regulation of the IL-6 and IL-8 induction in IBV-infected H1299 and Vero cells by the p38 MAPK and dual-specificity phosphatase 1 feedback loop was observed in our previous studies [44].

Here, we found that IBV-induced IL-8 expression was enhanced by overexpression of PKR and the constitutively active phosphomimetic mutant of eIF2α (S51D), but not by the overexpression of the PKR mutant K296P, wild type eIF2α, or eIF2α-S51A. IBV-induced ATF3 expression was also upregulated in cells transfected with PKR or S51D, suggesting effective activation of the PKR/eIF2α/ATF3 pathway by these constructs. It is interesting that induction of IL-8 and ATF3 was not upregulated in cells transfected with wild type eIF2α. In addition, the induction of IL-8 mRNA was shown to increase when eIF2α dephosphorylation was inhibited by the treatment with SAL, supporting that the phosphorylation status of eIF2α, but not its total protein level, was responsible for promoting the IBV-induced IL-8 expression. Alternatively, the ectopic eIF2α with a FLAG-tag may not be properly recognized by endogenous eIF2α kinases. The observation that the overexpressed phosphomimetic mutant S51D is functionally active would support this argument, as interactions with an eIF2α kinase are not required for the activity of the S51D construct. 

Apart from IBV, the activation of PKR/PERK–eIF2α by coronavirus infection has been extensively characterized in numerous studies. For example, significant phosphorylation of eIF2α and sustained translation suppression of host proteins were detected in cells infected with MHV starting from 8 h post infection [55]. Similarly, phosphorylation of PKR and eIF2α was observed in cells infected with SARS-CoV [28] and TGEV [56]. In our previous studies, we established the temporal activation kinetics of the PKR/PERK–eIF2α pathway in Vero and H1299 cells infected with IBV. As the focus of the current study is the regulatory function of ISR on cytokine induction, we have chosen the induction of ATF3 mRNA as a surrogate readout for the activation level of the PKR/PERK–eIF2α pathway.

One potential activating mechanism of eIF2α may be mediated by regulating the activity of NF-κB, a crucial transcription factor that regulates the expression of most proinflammatory cytokines [24]. The eIF2α-mediated translation shutoff may reduce the production of NF-κB inhibitor alpha (IκBα) protein, thereby releasing its inhibition of NF-κB. In fact, NF-κB-mediated IFN-I production in TGEV-infected cells was dependent on the activation of PERK and eIF2α [31]. Further studies focused on characterizing the regulation of NF-κB by ISR during coronavirus infection, as well as the roles of other eIF2α (such as HRI and GCN2) in this process, would be required.

Besides NF-κB, ISR may also regulate cytokine production via other mechanisms. Several genes that are normally repressed become preferentially translated under ISR, including activating transcription factor (ATF4), C/EBP-homologous protein (CHOP), and GADD34 (growth arrest and DNA damage-inducible protein 34). As a subunit of protein phosphatase 1, GADD34 promotes the dephosphorylation of eIF2α to revert the translation shutoff, thereby serving as a negative feedback regulator of ISR. In addition, GADD34 has been shown to participate in the innate immune response during viral infection. For example, GADD34 is essential for the production of IFN-β and IL-6 in cells infected with the Chikungunya virus or treated with polyI:C [57,58]. Mechanistically, eIF2α-mediated translation shutoff reduced the protein levels of negative regulators (such as IκBα) and potentiated the activation of the dsRNA–RLRs–IRF3/IRF7 innate immune pathway [59]. Importantly, transcription of GADD34 is synergistically activated by IRF3/IRF7 and ATF4, and the GADD34-mediated restoration of protein translation allowed for a pulse cycle of cytokine synthesis [59]. Previously we have shown that IBV infection activated the PERK–ATF4–CHOP pathway and induced the expression of GADD34 to maintain de novo protein synthesis in the infected cells [30]. It would be interesting to further investigate the role of GADD34 in coronavirus-induced cytokine production.

In this study, we show that the AP-1 family members, cFOS and cJUN, are distinctively involved in the IBV-induced IL-8 production. Manipulation of the cFOS expression on its own did not affect IL-8 expression, but its coexpression of cJUN further enhanced IBV-induced IL-8 levels. The different effects between cJUN and cFOS may lie on their differing activation mechanisms, as previously characterized in cells stimulated with stress and/or cytokines. The functional efficacy of cFOS is mainly driven by de novo protein synthesis, whereas cJUN is mainly activated by phosphorylation at the N-terminal Ser63 and Ser73 residues by JNK [60]. Consistently, our recent study has shown that in IBV-induced H1299 cells, cFOS mRNA was induced by ~500-fold while cJUN mRNA was induced by ~40-fold [45]. In addition, the basal level of cFOS protein was not detectable in the uninfected cells, but it rose to very high levels at the peak of IBV infection [45]. On the contrary, the basal level of the cJUN protein was relatively high in the uninfected cells, and only minor induction of its protein level was detected in the IBV-infected cells [37]. It is possible that IBV infection induced the mRNA and protein expression of cFOS to such saturating high levels, that the activating effect of cFOS on IL-8 induction became insensitive to its overexpression and knockdown. Use of a specific cFOS inhibitor, such as T-5224, a rationally designed small molecule that inhibits the DNA binding activity of cFOS/cJUN, would be of help in exploring this possibility further. In fact, when H1299 cells were treated with T-5224 at 12.5 µM in a previous study, IBV replication was not affected, but IBV-induced IL-8 expression was significantly reduced compared with the solvent-treated control [45]. It suggests that the DNA binding activity of cFOS is indeed required for IBV-induced IL-8 expression. Furthermore, two functional domains of cFOS, the basic domain (BD) and the leucine zipper domain (LZD), have been characterized. The LZD is essential for dimerization between cFOS and other AP-1 proteins, whereas the BD is required for both DNA-binding and cytosolic functions of cFOS [61]. We have previously shown that inhibition of the nuclear translocation of cFOS using a nuclear localization signal peptide (NLSP) resulted in the reduction of IBV viral protein synthesis and IBV-induced apoptosis [45]. It would be interesting to establish cFOS-knockout cell clones and use BD and LZD mutants to further investigate how the nuclear and cytoplasmic activities of cFOS would regulate cytokine production in coronavirus-infected cells.

Overexpression of cJUN significantly increased the IBV-induced IL-8 transcription; however, overexpression of the JNK upstream kinase MKK7 rendered only a limited effect. This may reflect the involvement of multiple upstream signaling pathways/kinases in the activation of cJUN in IBV-infected cells, in addition to the JNK pathway. In fact, cJUN and its upstream kinases themselves may have different or even opposite effects in cell signaling. For example, we have previously shown that JNK promotes IBV-induced apoptosis independently of cJUN [37]. Further experiments using cJUN mutants harboring mutations in the N-terminal phosphorylation sites, the LZD and the transactivation domains, would be useful to unravel its underlying mechanisms. A previous study has shown that IL-8 induced by SARS-CoV S protein is dependent on AP-1 activation but independent of NF-κB activation [50]. It would also be interesting to study the crosstalk between the AP-1, NF-κB, and ISR signaling pathways in the context of coronavirus infection. Finally, the SARS-CoV E protein harbors a PDZ-binding motif (PBM) that interacts with the host protein, syntenin. This interaction relocates syntenin to the cytoplasm, where it activates p38 to induce the expression of proinflammatory cytokines [62]. It would be interesting to see whether other viral factors directly or indirectly interact with ISR and/or AP-1 proteins, thereby modulating cytokine expression during coronavirus infection.

In this study, induction of IL-8 mRNA by IBV infection is consistently enhanced by the inhibition of eIF2α dephosphorylation and the overexpression of PKR, constitutively active eIF2α, cJUN, and MKK7; whereas IBV-induced IL-6 expression is less responsive to these treatments. The core promoter sequences of IL-6 and IL-8 both contain binding sites for AP-1, C/EBPβ, and NF-κB. However, the IL-6 promoter also contains a binding site for cAMP response element binding protein (CREB), which is absent in the IL-8 promoter. In addition, the distant enhancer sequences and post-transcriptional regulatory mechanisms also differ for the two cytokines/chemokines. It is possible that one or more factors essential for efficient IL-6 (but not IL-8) mRNA expression is missing or scarce in epithelial cell lines used in this study. This may explain why transcription of IL-8 is more pronounced and responsive for PKR/cJUN overexpression than IL-6 during IBV infection. In fact, differential regulation of the IL-6 and IL-8 induction in IBV-infected cells by the p38 MAPK and dual-specificity phosphatase 1 feedback loop was observed in our previous studies [44].

To conclude, we have demonstrated that both ISR and AP-1 proteins contributed significantly to the transcriptional induction of IL-8 in coronavirus-infected cells. This study adds to our understanding of the complex interactions between cellular stress pathways and innate immune response against coronavirus infection.

## 4. Materials and Methods

### 4.1. Cell Culture and Virus Infection

Vero cells were cultured in Dulbecco modified Eagle medium (DMEM, Gibco, Shanghai, China) supplemented with 5% fetal bovine serum (FBS) and 1% penicillin–streptomycin (Gibco). H1299 cells were cultured in RPMI1640 medium (Gibco) supplemented with 5% FBS and 1% penicillin–streptomycin (Gibco). All cells were grown in a 37 °C incubator supplied with 5% CO_2_.

The egg-adapted Beaudette strain of IBV (ATCC VR-22) was obtained from American Type Culture Collection (ATCC) and adapted to Vero cells as previously described [63]. This Vero-adapted strain was named IBV-p65 and the complete genome sequence was uploaded (accession No. DQ001339) [63,64]. The virulent strain DR13 of PEDV (PEDV-vDR13) was isolated in Korea in 1999 (accession No. JQ023162) as previously reported [65].

To prepare the virus stock, monolayers of Vero cells were infected with IBV-p65 or PEDV-vDR13 at an MOI of approximately 0.1 and cultured in plain DMEM at 37 °C until complete fusion of the entire monolayer was observed. After three freeze/thaw cycles, cell lysate was clarified by centrifugation at 1500× *g* at 4 °C for 30 min. The supernatant was aliquot and stored at −80 °C as virus stock. The titer of the virus stock was determined by plaque assays. The mock lysate was prepared by the same treatment of uninfected Vero cells. 

Unless stated otherwise, for IBV infection experiments in cultured cells, cells in a 12-well plate were first washed twice with serum-free medium. The cells were then infected with IBV or PEDV at MOI~2 or incubated with an equal volume of mock lysate. After 2 h of adsorption, the cells were washed twice and incubated in serum-free medium at 37 °C until they are harvested. Cell lysates and supernatant samples were harvested as stated below.

### 4.2. Antibodies, Chemicals, and Reagents

The antibodies against β-actin (#4967), cJUN(#9165), and cFOS(#2250) were purchased from Cell Signaling Technology, Shanghai, China. The antibody against IL-8 (#sc-32817) was purchased from Santa Cruz Biotechnology, Shanghai, China.The antibody against FLAG tag (#HT201-01) was purchased from Transgen Biotech, Beijing, China. The antiserum against IBV N protein were isolated from rabbits immunized with bacterial expressed fusion proteins as previously described [66]. Salubrinal (#HY-15486) is purchased from MedChemExpress (Shanghai, China) and dissolved in DMSO for a 50 mM stock solution. 

### 4.3. Plasmid Construction and Transfection

Unless stated otherwise, the vector for all overexpression plasmids was pXJ40-FLAG, in which expression of the FLAG-tagged transgene was driven by a CMV enhancer/promoter with a downstream rabbit beta globin intron, and terminated with a SV40 polyadenylation signal [67]. The plasmids pXJ40-FLAG-PKR, pXJ40-FLAG-PKR-K296R, pXJ40-FLAG-eIF2α, pXJ40-FLAG-eIF2α-S51A, pXJ40-FLAG-eIF2α-S51D, pXJ40-FLAG-MKK7, and pXJ40-FLAG-MKK7-K149M were cloned as previously described [30,37]. The plasmids pcDNA-FLAG-MKK7-JNK1 and pcDNA-FLAG-MKK7-JNK1(APF) were obtained from Addgene, Watertown, MA, USA [68].

The complementary DNA (cDNA) of human cJUN (RefSeq NM_002228) was amplified from total RNA of H1299 cells by reverse transcription-polymerase chain reaction (RT-PCR) using the forward primer CCC*GGATCC*ATGACTGCAAAGATGGAAACGACC and reverse primer CTT*GGTACC*TCAAAATGTTTGCAACTGCTGCG. The PCR product was digested and inserted into pXJ40-FLAG using the BamHI and KpnI sites for pXJ40-FLAG-cJUN. The dominant negative (DN) trans-activation mutant TAM-67 of cJUN was amplified using the same template and reverse primer, but with the forward primer CCC*GGATCC*ATGACTAGCCAGAACACGCTGCCC. The product was cloned similarly to generate pXJ40-FLAG-cJUN-DN.

The cDNA of human cFOS (RefSeq NM_005252) was amplified from total RNA of H1299 cells by RT-PCR using the forward primer ACCA*GGATCC*ATGATGTTCTCGGGCTTCAAC and reverse primer ATTA*CTCGAG*TCACAGGGCCAGCAGC. The PCR products were digested and inserted to pXJ40-FLAG using the BamHI and XhoI restriction sites for pXJ40-FLAG-cFOS.

Transfection of plasmids was performed using the TransIntro EL reagent (Transgen Biotech, Beijing, China) according to the manufacturer’s instruction. Briefly, cells were plated to a 12-well plate the day before transfection. For each well, 1.6 µg plasmid DNA and 2 µL TransIntro EL were diluted with 100 µL Opti-MEM (Gibco) and incubated for 20 min. The cells were replenished with 900 µL Opti-MEM containing 5% FBS, and the transfection mixture was added to each well dropwise. Virus infection was performed at 24–36 h post-transfection.

### 4.4. SDS-PAGE and Western Blot Analysis

To obtain whole-cell lysates for protein analysis, cells were harvested at the indicated time points using cell scrapers (Corning) and collected by centrifugation at 16,000× *g* for 1 min. The supernatant was discarded, and the cell pellet was lysed in 1× RIPA buffer (10 mM Tris-HCl pH 8.0, 140 mM NaCl, 0.1% SDS, 1% Triton X-100, 0.1% sodium deoxycholate, 1 mM EDTA, and 0.5 mM EGTA). After being clarified by centrifugation, the protein concentration of the cell lysate was determined. The cell lysate was then mixed with 5× Laemmli sample buffer (0.3125 M Tris-HCl pH 6.8, 10% SDS, 50% glycerol, 25% β-mercaptoethanol, and 0.025% bromophenol blue), boiled at 90 °C for 5 min, and centrifuged at 16,000× *g* for 5 min [69]. Equal amounts of protein samples were loaded to each well and separated by sodium dodecyl sulfate-polyacrylamide gel electrophoresis (SDS-PAGE) using the Bio-Rad Mini-PROTEAN Tetra cell system. The resolved proteins were then transferred to a 0.2 µm nitrocellulose membrane using the Bio-Rad Trans-Blot protein transfer system. To block off non-specific binding sites, the membrane was incubated with 5% skim milk in 1× TBST buffer (20 mM Tris-HCl pH 7.4, 150 mM NaCl, 0.1% Tween 20) at room temperature for 1 h. The membrane was then incubated with 1 µg/mL specific primary antibody dissolved in 1× TBST with 3% BSA (*w*/*v*) at 4 °C overnight. The membrane was washed three times with 1× TBST, and incubated with 1:10,000 diluted IRDye 800CW goat anti-Rabbit or 680RD goat anti-mouse IgG secondary antibodies (Licor) at room temperature for 2 h. The membrane was washed three times with 1× TBST, and fluorescence imaging was performed using the Azure c600 Imager according to the manufacturer’s instruction. All experiments were repeated for at least three times with similar results, and one of the representative results is shown.

### 4.5. RNA Extraction and RT-qPCR Analysis

Total RNA was extracted using the TRIzol reagent (Invitrogen, Shanghai, China) according to the manufacturer’s instructions. Briefly, cells were lysed with 1 mL TRIzol per 10 cm² effective growth area, and the lysates were vigorously mixed with one-fifth volume of chloroform. The mixture was then centrifuged at 12,000× *g* at 4 °C for 15 min, and the aqueous phase was mixed with an equal volume of isopropanol. The RNA was precipitated by centrifugation at 12,000× *g* at 4 °C for 15 min, washed twice with 70% ethanol, and dissolved in 30–50 µL RNase-free water. The total RNA was reverse transcribed using the FastKing gDNA Dispelling RT SuperMix kit (Tiangen, Beijing, China) according to the manufacturer’s instructions. Briefly, 2 µg total RNA was mixed with 4 µL 5× FastKing-RT SuperMix (containing RT enzyme, RNase inhibitor, random primers, oligo dT primer, dNTP, and reaction buffer) in a 20 µL reaction mixture. Using a thermo cycler, reverse transcription was performed at 42 °C for 15 min and the RT enzyme was then inactivated at 95 °C for 3 min. The cDNA was then diluted 20-fold with RNase-free water for quantitative PCR (qPCR) analysis, using the Talent qPCR PreMix SYBR Green kit (Tiangen) according to the manufacturer’s instructions. Briefly, 8.4 µL diluted cDNA was mixed with 10 µL 2× qPCR PreMix, 0.4 µL 50× ROX, 0.6 µL 10 µM forward primer, and 0.6 µL 10 µM reverse primer for a 20 µL reaction mixture. The qPCR analysis was performed using a QuantStudio 3 Real-Time PCR System (Applied Biosystems, Shanghai, China). The standard protocol included enzyme activation at 50 °C for 3 min, initial denaturation at 95 °C for 3 min, followed by 40 cycles of denaturing (95 °C, 5 s) and annealing/extension (60 °C, 30 s) with fluorescent acquisition at the end of each cycle. The results obtained were in the form of cycle threshold (CT) values. Using the ΔΔCT method, the relative abundance of a transcript was calculated using GAPDH as an internal control and normalized to the respective control sample. For IBV genomic RNA, a standard curve based on pKT-IBVcDNA-A was used to estimate the copy number of IBV genome in the sample. All experiments were repeated at least three times with similar results, and one of the representative results is shown.

The following qPCR primer pairs were used: IBV genomic RNA (gRNA) GTTCTCGCATAAGGTCGGCTA and GCTCACTAAACACCACCAGAAC, PEDV gRNA AGTAGCCATCGCAAGTGCTG and AACCGGAGGAAGGCTGTTTG, GAPDH CTGGGCTACACTGAGCACC and AAGTGGTCGTTGAGGGCAATG, IL-8 (Vero) AAGACGTACTCCAAACCTATCCAC and TCTGTATTGACGCAGTGTGGTC, IL-6 (Vero) GTGCAAATGAGTACAAAAGTCCTGA and GTTCTGCGCCTGCAGCTTC, ATF3 (Vero) CTCTGCGCTGGAGTCAGTCA and TTCTTTCTCGCCGCCTCTTTTT, IL-8 (human) ATAAAGACATACTCCAAACCTTTCCAC and AAGCTTTACAATAATTTCTGTGTTGGC, IL-6 (H1299) GTGCAGATGAGTACAAAAGTCCTGA and GTTCTGTGCCTGCAGCTTC, ATF3 (human) CCTCTGCGCTGGAATCAGTC and TTCTTTCTCGTCGCCTCTTTTT, cJUN (human) AACAGGTGGCACAGCTTAAAC and CAACTGCTGCGTTAGCATGAG, and cFOS (human) GGGGCAAGGTGGAACAGTTAT and CCGCTTGGAGTGTATCAGTCA. 

### 4.6. Statistical Analysis

The two-way ANOVA method was used to analyze the significant difference between the indicated sample and the respective control sample. Significance levels were presented by the *p* value (ns, non-significant; *, *p* < 0.05; **, *p* < 0.01; ***, *p* < 0.001). 

## Data Availability

The raw data supporting the conclusions of this article will be made available by the authors, without undue reservation.
